# A novel therapeutic approach for macrophage actıvatıon syndrome due to SoJIA

**DOI:** 10.1186/1546-0096-9-S1-P104

**Published:** 2011-09-14

**Authors:** Nuray Aktay Ayaz, Esra Şevketoğlu, Sait Serdar Oral, Tuğba Kontbay, Deniz Aybar, Sami Hatipoğlu, Gönül Aydoğan

**Affiliations:** 1 SB Bakırköy Doğumevi Kadın Doğum ve Çocuk Hastalıkları Eğitim ve Araştırma Hastanesi; 2 SB Bakırköy Dr Sadi Konuk Eğitim ve Araştırma Hastanesi

## Case

A 12 year-old boy who had been diagnosed as juvenile idiopathic arthritis (JIA) nine years ago, had attended to our hospital with the complaints of high fever, arthralgia and abdominal pain. He was febrile with salmon colored maculopapular rash all over the body. He was evaluated for infections and malignancy. He was diagnosed as systemic onset JIA (SoJIA). Pulse steroid therapy was started. He was fine at the second day of pulse therapy, at the 3rd day his rash recurred and he became febrile all day long. We detected pancytopenia, low ESR, hyperferritinemia, high D-dimer, low fibrinogen and hypertriglyceridemia. His pulse was prolonged to 5 days and cyclosporin (5 mg/kg) was commenced with the diagnosis of macrophage activation syndrome. He became worse clinically and his ferritin levels continued to rise. Intraveneous immunoglobulin (2 gr/day) was added but he was resistant. Hemophagocytic lymphohistiocytosis protocol was started. His ferritin was elevated until 135.000 ng/ml. Etanercept treatment (o.4 mg/kg twice a week) was introduced. With renewal of the laboratory findings we decided to start hemofiltration and plasmapheresis in order to deecrease the cytokine overload. He was taken under continious hemofiltration and plasmapheresis. After 22 days in the PICU his ferritin levels decreased to 1500 ng/ml. He had arthralgias and elevation of acute phase response once more, this picture was attributed to activation of SoJIA and metotrexate therapy was started. We presented our very resistant case to point out the success of a novel therapeutic approach that is plasmapheresis and hemofiltration.

